# Supplementary material for: Ocean acidification decreases grazing pressure but alters morphological structure in a dominant coastal seaweed
Source: PLoS One. 2021 Jan 28;16(1):e0245017. doi: 10.1371/journal.pone.0245017 (PMC7842949; doi:10.1371/journal.pone.0245017)
Supplement: Attachment — Submitted filename: Kinnby et al PlosONE2020.docx [file pone.0245017.s002.docx]

**Increased thallus area and decreased breaking strength in a habitat forming seaweed** **under ocean acidification**

Alexandra Kinnby^1^, Joel C.B. White^1^, Gunilla B. Toth^1^, Henrik Pavia^1^

^1^Department of Marine Sciences, University of Gothenburg, Tjärnö Marine Laboratory, Strömstad, 452 96, Sweden

*Corresponding author, e-mail: [alexandra.kinnby@marine.gu.se](mailto:alexandra.kinnby@marine.gu.se) (AK)

**ABSTRACT**

Ocean acidification driven by anthropogenic climate change is projected to cause a drop in pH of 0.4 units in coastal shallow waters by the year 2100. Previous studies have shown that seaweeds grown under such conditions may alter their growth and photosynthetic capacity, and that calcified herbivores struggle to maintain shell density. It is not clear how such alterations might impact interactions between seaweed and herbivores, *e.g.* through changes in feeding rates, nutritional value, or defense levels. Changes in seaweeds are particularly important for coastal food webs, as they are key primary producers and often habitat-forming species.

We cultured the habitat-forming brown seaweed *Fucus vesiculosus* for 30 days in projected future pCO_2_ (1100 ppm) with genetically identical controls in ambient pCO_2_ (400 ppm). Thereafter the macroalgae were exposed to grazing by *Littorina littorea*, acclimated to the relevant pCO_2_-treatment. We found increased growth (measured as surface area increase), decreased tissue strength in a pulling to break test, and decreased chemical defense (phlorotannins) levels in seaweeds exposed to high CO_2_-levels. The herbivores exposed to elevated CO_2_-levels showed improved condition index, decreased consumption, but no significant change in feeding preference.

Fucoid seaweeds such as *F. vesiculosus* play important ecological roles in coastal habitats and are often foundation species, with a key role for ecosystem structure and function. The change in surface area and associated decrease in breaking force, as demonstrated by our results, indicate that *F. vesiculosus* grown under elevated levels of pCO_2_ may acquire an altered morphology with increased drag and reduced tissue strength. This, together with increased wave energy in coastal ecosystems due to climate change, could have detrimental effects by reducing both habitat and food available for herbivores.

**Keywords:** *Fucus vesiculosus; Littorina littorea;* Grazer seaweed interaction; Climate change; Brown seaweed

**INTRODUCTION**

Ocean acidification (OA) is the drop in pH caused by the absorption of atmospheric CO_2_ into the surface of the oceans [1]. The majority of dissolved CO_2_ concentrates above the thermocline, generating an estimated drop in pH to 7.7 [2] or 0.4 units [1,3,4] by year 2100 in surface and coastal waters [5]. Thus, coastal ecosystems and the organisms that live there are expected to be most impacted by ocean acidification. Seaweeds are key habitat-forming primary producers that support high biodiversity in coastal areas [6], and therefore their response to ocean acidification may have impacts throughout the ecosystem. Seaweeds use CO_2_ for carbon fixation and growth, and may therefore benefit from the increase in available carbon caused by ocean acidification [7]. A growing number of studies have, however, shown that ocean acidification can have positive, neutral, or negative direct effects on basic performance traits such as growth and photosynthesis of seaweeds [*e.g.* 7,8], and these effects may differ between life stages of a species, as well as between closely related species [9–11].

Aside from effects on basic performance traits, ocean acidification can also impact both primary and secondary metabolism in seaweeds, sometimes resulting in higher carbon to nitrogen (C:N) and carbon to phosphorous (C:P) ratios [12, 13 but see 14]. Increase in carbon availability for seaweeds generally results in decreased protein content [*e.g.* 15–19], and either increased [17,18] or decreased [20] levels of fatty acids. Furthermore, the content of secondary metabolites, such as the grazing deterrent dimethylsulfoniopropionate (DMSP) in green seaweeds, has been shown to increase in response to elevated pCO_2_ levels [15]. In brown seaweeds, phlorotannins (polyphenolic compounds) are ubiquitous metabolites that can occur in high concentrations, especially in fucoid species (*Fucales*). Phlorotannins have multiple functions *e.g.* as defense against UV-radiation and defense compounds against gastropod grazing [21,22]. To our knowledge, only two studies have investigated the effects of ocean acidification on phlorotannin production in brown seaweeds, with mixed results [9,11]. Olischläger *et al*. [9] found no effect on phlorotannin production in the kelp *Laminaria hyperborea* when grown under 700 ppm pCO_2_, while Swanson & Fox [11] found increased phlorotannin production in *Saccharina latissima* but not *Nereocystis leutkeana* when exposed to 3000 ppm pCO_2_.

The nutritional and defensive characteristics of seaweeds are critical traits in ecological interactions since they affect the growth and fecundity of herbivores [23,24]. Therefore, apart from direct effects on the physiology and biochemical content, ocean acidification may also have indirect effects on macroalgae through interactions with grazers. A decrease in the nutritional value and increase in deterrent defense metabolites under ocean acidification may lower the palatability of macroalgae to grazers [*e.g.* 16,25–27]. This may, however, also lead to an increase in the *per capita* grazing pressure through compensatory grazing if less nutritious food is available [*e.g.* 16]. Grazing may also be altered by direct effects of ocean acidification on the herbivore, *e.g.* through changes in respiration or behavior [*e.g.* 28,29]. Bibby *et al*. [28] showed that the snail *Littorina littorea* had noticeable reductions in both metabolic rate and induced defense (shell formation), which increased the avoidance behavior of the snails and could in turn affect their interactions with other species. Additionally, Young *et al.* [29] found that the grazing rate decreased when a snail (*Lacuna vincta*) was exposed to elevated pCO_2_, irrespective of the effects of pCO_2_ on the seaweeds (*Ulva* spp.) that the snail was grazing on.

In temporal coastal ecosystems, fucoids are dominant habitat-forming seaweeds that provide shelter, habitat, and food for other organisms [30]. Fucoid presence is associated with a local increase in species abundance and diversity [31], but there is no consensus how ocean acidification will affect the adult stage of associated species [but see *e.g.* 32 for effects on early life-stages]. Since many fucoids have an active uptake of bicarbonate [33], which is very abundant in seawater, it has been suggested that they would not increase growth in response to increased pCO_2_ [34]. To our knowledge, there are only two previous studies that investigate potential indirect effects of ocean acidification on fucoids through changes in interactions with herbivores [35,36]. In one study, the consumption of the herbivore *Littorina obtusata* on the seaweed *F. vesiculosus* was somewhat, but not statistically significantly, increased under ocean acidification conditions [36], while in another the interaction between *F. vesiculosus* and herbivores was not affected by changes in pH [35]. This relative lack of literature is surprising, considering that fucoids are common and important species in the upper part of the photic zone where ocean acidification is predicted to have the largest impact. Given the high phlorotannin content found in many fucoids, the effect of ocean acidification on phlorotannin production may also alter the interaction between seaweed and herbivore, but this has, to our knowledge, not yet been investigated.

The overall aim of the present study was to examine potential direct effects of ocean acidification on the common fucoid *F. vesiculosus*, as well as indirect effects on the seaweed through changes in its interactions with the common gastropod grazer *L. littorea*. We conducted manipulative experiments to determine how the growth rate, photosynthesis, carbon and nitrogen content, as well as chemical defenses (phlorotannin content), and breaking strength of *F. vesiculosus* will be affected by increasing CO_2_ levels in the future. Furthermore, we also tested the effect of elevated pCO_2_ on consumption, feeding preference, and condition index of *L. littorea*.

**MATERIALS AND METHODS**

*Experimental design*

Sixty individuals of *F. vesiculosus* were collected from the west coast of Sweden, in July 2018 and kept at Tjärnö Marine Laboratory (TML, 58°52’36.4”N 11°6’42.84”E) under ambient conditions for 7 days to acclimatize to laboratory conditions. *F. vesiculosus* in western Sweden spends much more time submerged than in other parts of its range due to the extremely small tidal range in the area, hence the algae were kept under water throughout the experiment. After the acclimation period, each seaweed was split into one experimental thallus and one control thallus, placed in separate 1 L aquaria with constant seawater flow from header tanks. Control thalli were maintained at ambient pCO_2_ (400 ppm) while experimental thalli were exposed to gradually increasing (~100 ppm/day) CO_2_ until a pCO_2_ of 1100 ppm was reached 7 days later [ corresponding to the projected value at the end of this century 37]. 360 individuals of *L. littorea* were also collected and exposed to the same conditions as the seaweed, *i.e*. 180 snails were exposed to ambient water and 180 snails were exposed to treatment water in separate tanks from the seaweed thalli (these snails were used in a grazing experiment described below). The header tanks were aerated with either ambient atmospheric air (pCO_2_ of 400 ppm) or CO_2_-enriched air controlled by solenoid valves and pH-computers (Aqua Medic) to provide a final pCO_2_ of 1100 ppm. The pCO_2_ was monitored with LI-850 CO_2_/H_2_O Gas Analyzer (Li-COR). Filtered seawater flow was constant at 0.3 L/min in each aquarium throughout the experiment. Salinity, temperature, pCO_2_, and pH were measured in the 1 L aquaria and alkalinity was calculated with CO2calc [38; Table 1].

**Table 1.** Average measured partial pressure of CO_2_ (pCO_2_), pH, salinity, and temperature for the experimental treatments. Total alkalinity (A_T_) was estimated from salinity and pCO_2_ measurements.

The seaweed thalli were weighed and photographed (for area measurements) at the beginning and end of the 30-day experiment. The efficiency of photosystems I and II (Fv/Fm and P- index) were measured in the new tissue formed during the experiment. The tissue was dark-adapted for 10 minutes, the fiber optics were held at a fixed 10 mm distance from the algae, and measurements were taken with a PAM (pulse amplitude-modulated fluorometer; Walz, Effeltrich, Germany). Breaking strength was measured by securing the seaweed to a dynamometer and increasing the strain until the thallus broke. Following this, apical tissue samples were frozen (-60°C) for further nutritional and phlorotannin analysis (see below).

*Phlorotannin analysis*

For phlorotannin analysis, the frozen samples were freeze-dried, homogenized to a fine powder, and 10 mg of each sample was extracted in 60% acetone. Total phlorotannin content was quantified colorimetrically using the Folin-Ciocalteus method [39], with phloroglucinol (1,3,5-trihydroxybenzene, art. 7069; Merck, Darmstadt, Germany) as standard.

*Elemental analysis*

For the determination of carbon (C) and nitrogen (N) content the frozen seaweed tissue was freeze-dried and homogenized to a fine powder and weighed to the nearest 0.01 mg. The total tissue C and N content, as well as δ^13^C and δ^15^N of the samples were analyzed with an elemental analyzer (ANCA-GSL, Sercon Ltd., Crewe, UK) coupled to an isotope ratio mass spectrometer (20-22, Sercon Ltd., Crewe, UK).

*Consumption and feeding preference of* Littorina littorea

The palatability of the *F. vesiculosus* thalli grown in ambient and elevated pCO_2_ during 30 days was measured in two-choice feeding trials using *L. littorea* as the grazer. The feeding experiment was performed using a total of 120 containers (200 mL) with constant seawater flow of ambient pCO_2_ (400 ppm). In each container two similarly sized apical pieces of *F. vesiculosus* were placed (0.50 ± 0.0017 g mean ± SE), one piece from the ambient pCO_2_ treatment and one from the high pCO_2_ treatment; as both pieces came from the same thallus they were genetically identical. Six individuals of *L. littorea*, exposed to either ambient or elevated pCO_2_ were placed in half of the containers (n=30, *i.e.* a total of 60 containers with herbivores). To control for autogenic changes in mass (growth) during the experiment that was not caused by the grazing of the snails, each container with seaweed pieces and herbivores were paired with a control container without herbivores once again containing the same genetic individual of seaweed. The wet weight of all seaweed pieces was determined at the start and at the end of the 24-hour experiment by using a standard blotting procedure, and the wet-weight change of each seaweed piece was calculated by subtracting the weight at the end of the experiment from the starting weight. The consumption of the snails exposed to different pCO_2_-levels was determined by calculating the total change in weight between initial and post-grazing weights for each container and subtracting the weight change in the autogenic control containers. To study feeding preference of herbivores exposed to different pCO_2_-levels, the difference between weight changes of the two seaweed pieces in each container was calculated by subtracting the wet weight change of the seaweed exposed to high pCO_2_ from the weight change of the seaweed piece exposed to ambient pCO_2_ [40].

*Condition index for* Littorina littorea

Following the feeding preference experiment all snails were euthanized by freezing at -20°C. To assess whether the elevated pCO_2_ treatment had affected the physiological status of the snails a condition index was calculated [36,41]. Snails were thawed and weighed whole; following this the shell was weighed alone. The dry weight of the soft tissue was obtained by weighing the body after drying for 48 hours at 50°C. The condition index was derived from the weights according to the following formula:

$$CI=(100*drytissueweight)/(wholeweight-shellweight)$$

*Data analysis*

The seaweed response variables, *i.e*. growth (% increase in area and weight), breaking strength, efficiency of photosystem I & II (Fv/Fm and P-index), as well as phlorotannin, and nutritional content were all statistically analyzed with paired t-tests to determine if there was a significant difference between the seaweed in ambient and elevated levels of pCO_2_. Paired t-tests were used because every treatment thallus was paired with a genetically identical control thallus. To investigate if there was a difference in condition index between the snails exposed to ambient and treatment water a t-test was performed. For the condition index the assumptions for homogenous variances were violated hence a Kolmogorov-Smirnov test was run. The consumption of herbivores exposed to ambient and elevated pCO_2_ was analyzed with a t-test. Preference for seaweed grown under the different pCO_2_ conditions was evaluated by comparing the difference in weight change between the seaweed pieces kept with the herbivores and their respective autogenic controls with two separate paired t-tests; one each for herbivores exposed to ambient and elevated pCO_2_. A significantly lower difference in wet weight change for seaweed pieces kept with herbivores compared to autogenic controls indicated a preference for feeding on the control seaweed [40]. All analyses were performed in RStudio (version 1.0.136).

**RESULTS**

The seaweed thalli exposed to elevated levels of pCO_2_ grew significantly more than the thalli exposed to ambient pCO_2_ when growth was measured as increase in surface area of the seaweed (Fig. 1a; Table 2). On average, growth rates under elevated pCO_2_ were 65% higher than growth under ambient conditions. However, thallus weight did not differ significantly between the two treatments (Fig 1b; Table 2). We found a significant difference in the force needed to break the seaweed tissue in the control group compared to the seaweeds exposed to elevated pCO_2_; thalli from the treatment group were 57% weaker than those in the control group (Fig 1c; Table 2). We found no statistically significant differences in the efficiency of photosystem I and II measured as Fv/Fm and P-index (Fig. 1d, e; Table 2).

**Figure 1.** Effects on response variables of *Fucus vesiculosus* grown under ambient (400 ppm) and elevated (1100 ppm) pCO_2_ for 30 days. Values are means ± 95% CI, n=60 for a-j and n=10 for k. Response variables measured as a) Growth measured % increase in area (n=60), b) growth measured as % increase in weight (n=60), c) breaking force (N) (n=20), d) efficiency of photosystem I (Fv/Fm) (n=60), e) efficiency of photosystem II (P index) (n=60), f) Phlorotannin content (%dw) (n=60), g) %Nitrogen (n=60), h) δ^15^N (n=60), i) C:N ratio (n=60), j) %Carbon (n=60), and k) δ13C (n=60).

**Table 2.** Summary of effects of ambient (400 ppm) and elevated (1100 ppm) levels of pCO_2_ on *Fucus vesiculosus* measured as ten responses. P-values and corresponding t-values and degrees of freedom of paired t-tests are reported for the analyses of all response variables as well as means and 95% confidence intervals. Values in bold denote statistically significant values.

| **Response variable** | **p-value** | **t-value** | **Df** | **Mean (400ppm)** | **95%CI (400ppm)** | **Mean (1100ppm)** | **95%CI (1100ppm)** |
| --- | --- | --- | --- | --- | --- | --- | --- |
| Growth: area (%) | **9.2e-07** | -5.48 | 59 | 147.5 | 6.54 | 180.1 | 11.47 |
| Growth: weight (%) | 0.767 | -0.30 | 59 | 136.0 | 5.91 | 136.7 | 4.68 |
| Breaking force (N) | **0.032** | 2.33 | 18 | 1.36 | 0.54 | 0.59 | 0.27 |
| Photosystem I (Fv/Fm) | 0.407 | -0.83 | 59 | 0.695 | 0.011 | 0.701 | 0.007 |
| Photosystem II (P index) | 0.147 | -1.47 | 59 | 1.5 | 0.20 | 1.7 | 0.19 |
| Phlorotannin content | **0.030** | 2.22 | 59 | 10.6 | 0.53 | 10.3 | 0.57 |
| % Nitrogen | 0.379 | 0.89 | 59 | 1.32 | 0.055 | 1.29 | 0.066 |
| % Carbon | 0.865 | 0.17 | 59 | 36.04 | 0.43 | 36.00 | 0.37 |
| C:N | 0.236 | -1.20 | 59 | 28.2 | 1.44 | 29.3 | 1.80 |
| δ^13^C | **4.762e-09** | 6.85 | 59 | -11.77 | 0.32 | -13.33 | 0.41 |
| δ^15^N | 0.315 | -1.01 | 59 | 6.51 | 0.14 | 6.57 | 0.13 |

The results of chemical analyses showed that there was a statistically significant decrease in phlorotannin content between the tips of thalli exposed to elevated levels of pCO_2_ and those being exposed to ambient levels (Fig. 1f; Table 2). There were no statistically significant differences in C or N tissue content, nor in the C:N ratio. The stable carbon isotope (δ^13^C) content of *F. vesiculosus* was significantly reduced when exposed to elevated pCO_2_. The δ^13^C values decreased to -13 % under elevated pCO_2_ levels. Tissue δ^15^N was not significantly changed when exposed to elevated levels of pCO_2_ (Fig. 1g-k; Table 2).

The mean condition index of *L. littorea* was 28.9% higher in snails that had been exposed to high pCO_2_-levels than those exposed to ambient pCO_2_-levels (K-S test, D=0.32, p=2.973e-08; Fig. 2). Despite having a higher condition index, the herbivores exposed to high pCO_2_ levels consumed 37.5% less than the snails exposed to ambient pCO_2_ (t-test, t_58_=2.67, p=0.0098, Fig. 2). However, the herbivores did not show a statistically significant difference in preference based on the experimental treatment of the seaweed, regardless if the herbivores had been exposed to ambient pCO_2_ (paired t-test, t_29_=-0.517, p=0.609) or elevated pCO_2_ (paired t-test, t_29_=-0.584, p=0.564).

**Figure 2.** a) Consumption of the alga *Fucus vesiculosus* by the gastropod *Littorina littorea*, exposed to ambient (400 ppm) and elevated (1100 ppm) pCO_2_ for 30 days. b) Condition index for individuals of *L. littorea* following the grazing experiment. Values are means ± 95% CI.

**DISCUSSION**

Seaweeds play important ecological roles in coastal habitats and are often foundation species, with a key role for ecosystem structure and function. Hence, it is important to understand how seaweeds will be directly affected by changes in their environment, and also if these changes will alter seaweed interactions with other species. Here, we show that elevated pCO_2_-levels increased the thallus area, decreased the phlorotannin content, and reduced the breaking strength of *F. vesiculosus*. We also show that there was no significant treatment effect on the palatability of *F. vesiculosus* thalli*,* but that the consumption by the grazer *L. littorea* decreased, while the condition index of the snails increased under exposure to elevated levels of pCO_2_.

Increased pCO_2_ had no significant effect on the weight change of *F.* *vesiculosus*, but significantly increased the thallus surface area*.* Previous studies with *F. vesiculosus* have reported unaltered [42,43] or reduced [25] growth, measured as wet weight, under elevated pCO_2_-levels. Graiff *et al*. [44], however, reported a tendency (not statistically significant) of higher growth of *F. vesiculosus,* measured both as wet weight and length in apical tips, at high pCO_2_ levels. These different experimental results suggest that other factors interact with pCO_2_ to determine growth, *e.g.* seasonality or the life-cycle stage (age) of the seaweed, or genetic differences due to local adaptation among the populations used in the experiments. Such genetic differences in phlorotannin production and growth was recently demonstrated among *F. vesiculosus* populations at distances less than 100 km [45].

The combination of a significantly larger thallus with no effect on the weight of *F. vesiculosus* under enhanced CO_2_ conditions found in the present study strongly indicates a decrease in tissue density. This interpretation is corroborated by the drastic (57%) decrease in breaking strength of the thallus. To our knowledge, this is the first time such an effect of increased pCO_2_ levels is reported for seaweeds, and it parallels findings in terrestrial plants. For example, Pretzsch *et al.* [46] documented an increase in growth rate, attributed to increasing CO_2_ levels, among tree species in central Europe between 1960 and 2014. They also showed that this increase in growth was coupled with a decrease in tissue density and/or strength. This leaves forests more vulnerable to the increased weather variability that is also associated with climate change [47]. Our results suggest that similar effects may also be present in coastal marine systems. The reduced breaking strength could make seaweeds more vulnerable to storms and wave action, which are projected to become more frequent as the climate changes [48,49].

There were no significant changes in either the efficiency of photosystem I (Fv/Fm) or photosystem II (P-index) in response to elevated pCO_2_ in our experiment. This indicates that *F. vesiculosus* does not increase photosynthesis when exposed to higher pCO_2_, which follows the findings of Fernández and colleagues [50] who demonstrated that the increased carbon availability from increased pCO_2_-levels had no effect on photosynthesis because most algae are not carbon-limited. Seaweeds in general acquire carbon by passive diffusion of CO_2_ and active transportation of bicarbonate; as the concentration of CO_2_ rises the amount of passively diffusing CO_2_ also rises, reducing the seaweed’s reliance on active transport proteins to RuBisCo [51]. *Fucus* spp. are more resistant to changes in carbon concentrations because they use two parallel CO_2_ pathways for photosynthesis, both directly taking up carbon from their environment and also storing it as an intermediate for use when other carbon in less available [52]. This coincides with the significant decrease in tissue δ^13^C, which indicates a transition away from active intake of bicarbonate towards passive uptake of CO_2_, which has been previously documented in both *Gracillaria* sp. and *Ulva* sp. [51]. This carbon capture strategy by *F. vesiculosus* exhibits characteristics from C_3_, C_4_, and CAM plants in terrestrial systems; intertidal *F. vesiculosus* has been shown to use this method to continue photosynthesis when they exhaust the CO_2_ available to them while they are exposed to air [52].

In terrestrial plants, increased atmospheric CO_2_ has been shown to increase C:N ratios as well as accumulate phenolic compounds, such as tannins, affecting the consumption and growth rates of grazers [53,54]. However, we found no significant differences in C or N tissue content, nor in the C:N ratio. In contrast, Gutow *et al*. [25] showed high levels of pCO_2_ (700 ppm) decreased the C:N ratio of *F. vesiculosus*. Studies on the effects of increased CO_2_ on phenolic compounds in marine macrophytes are few and only one study on kelp species found that elevated CO_2_ leads to increased levels of phlorotannins [11]. By contrast, marine vascular plants have been shown to reduce phenolic acid production under increased CO_2_ conditions [55], which aligns with our results on *F. vesiculosus* showing slightly lower phlorotannin content in apical tips exposed to high pCO_2_-levels.

Despite finding a somewhat lower phlorotannin content in seaweeds exposed to elevated pCO_2,_ we did not find a difference in grazing preference of the gastropod *L. littorea* between seaweeds from the different treatments. However, snails exposed to elevated pCO_2_ generally consumed less than those exposed to ambient conditions, regardless of which food type they were offered. Reduced consumption by snails at increased pCO_2_ levels could indicate easier ingestion and digestion of food, or decreased activity of the grazer (and therefore decreased caloric requirements). The snails exposed to elevated levels of pCO_2_ had a higher condition index than snails exposed to ambient conditions. This combination of results is surprising, as a decreased consumption would be expected to result in a drop in condition index. Increased condition index, *i.e.* a higher dry to wet weight ratio of the soft tissue, could indicate more accumulation of tissue, *i.e.* increased growth, but also possibly failure to osmoregulate or other associated physiological problems. In summary, the results from the feeding experiment in the present study suggest that there are direct effects of increased pCO_2_ on herbivores and their consumption of seaweeds, but any indirect effects mediated by changes the palatability of the seaweeds are harder to discern.

In conclusion, our study shows that under ocean acidification conditions the habitat forming seaweed *F.* *vesiculosus* increases growth by thallus area, reduces reliance on active carbon uptake, shows a slight decrease in phlorotannin content and a drastic reduction in breaking strength. At the same time the herbivore *L.* *littorea* seems to tolerate increased pCO_2_, with an increased condition index even as they reduce their consumption of seaweeds. The reduced consumption rate of the herbivore suggests that the seaweed could gain some ecological benefits under ocean acidification. However, our most striking and unanticipated finding – that the seaweed could become much more vulnerable to physical forces under ocean acidification because of a significantly reduced breaking strength – could result in substantial loss of seaweed biomass due to increased storm events that are associated with climate change. This might in turn have implications for the future community structure of shallow coastal areas under ocean acidification.

**ACKNOWLEDGEMENTS**

We are grateful to Gunnar Cervin (University of Gothenburg) for help with the experiments and Kerstin Johannesson (University of Gothenburg) for valuable comments on the manuscript.

**References**

1.	K. C, E. WM. Anthropogenic carbon and ocean pH. Nature. 2003;425: 2003.

2.	Stocker TF, Qin D, Plattner GK, Tignor MMB, Allen SK, Boschung J, et al. Climate change 2013 the physical science basis: Working Group I contribution to the fifth assessment report of the intergovernmental panel on climate change. Clim Chang 2013 Phys Sci Basis Work Gr I Contrib to Fifth Assess Rep Intergov Panel Clim Chang. 2013;9781107057: 1–1535. doi:10.1017/CBO9781107415324

3.	Feely RA, Sabine CL, Lee K, Berelson W, Kleypas J, Fabry VJ, et al. Impact of anthropogenic CO2 on the CaCO3 system in the oceans. Science (80- ). 2004;305: 362–366. doi:10.1126/science.1097329

4.	Orr JC, Fabry VJ, Aumont O, Bopp L, Doney SC, Feely RA, et al. Anthropogenic ocean acidification over the twenty-first century and its impact on calcifying organisms. Nature. 2005;437: 681–686. doi:10.1038/nature04095

5.	Sabine CL, Feely RA, Gruber N, Key RM, Lee K, Bullister JL, et al. The Oceanic Sink for Anthropogenic CO 2. Science (80- ). 2004;305: 367–371.

6.	Wernberg T, Krumhansl K, Filbee-Dexter K, Pedersen MF. Status and trends for the world’s kelp forests. Second Edi. World Seas: An Environmental Evaluation Volume III: Ecological Issues and Environmental Impacts. Elsevier Ltd.; 2018. doi:10.1016/B978-0-12-805052-1.00003-6

7.	Koch M, Bowes G, Ross C, Zhang XH. Climate change and ocean acidification effects on seagrasses and marine macroalgae. Glob Chang Biol. 2013;19: 103–132. doi:10.1111/j.1365-2486.2012.02791.x

8.	van der Loos LM, Schmid M, Leal PP, McGraw CM, Britton D, Revill AT, et al. Responses of macroalgae to CO2 enrichment cannot be inferred solely from their inorganic carbon uptake strategy. Ecol Evol. 2019;9: 125–140. doi:10.1002/ece3.4679

9.	Olischläger M, Bartsch I, Gutow L, Wiencke C. Effects of ocean acidification on different life-cycle stages of the kelp Laminaria hyperborea (Phaeophyceae). Bot Mar. 2012;55: 511–525. doi:10.1515/bot-2012-0163

10.	Langer G, Nehrke G, Probert I, Ly J, Ziveri P. Strain-specific responses of Emiliania huxleyi to changing seawater carbonate chemistry. Biogeosciences. 2009;6: 2637–2646. doi:10.5194/bg-6-2637-2009

11.	Swanson AK, Fox CH. Altered kelp (Laminariales) phlorotannins and growth under elevated carbon dioxide and ultraviolet-B treatments can influence associated intertidal food webs. Glob Chang Biol. 2007;13: 1696–1709. doi:10.1111/j.1365-2486.2007.01384.x

12.	Urabe J, Togari J, Elser JJ. Stoichiometric impacts of increased carbon dioxide on a planktonic herbivore. Glob Chang Biol. 2003;9: 818–825. doi:10.1046/j.1365-2486.2003.00634.x

13.	Van De Waal DB, Verschoor AM, Verspagen JMH, Van Donk E, Huisman J. Climate-driven changes in the ecological stoichiometry of aquatic ecosystems. Front Ecol Environ. 2010;8: 145–152. doi:10.1890/080178

14.	Leung JYS, Nagelkerken I, Russell BD, Ferreira CM, Connell SD. Boosted nutritional quality of food by CO2 enrichment fails to offset energy demand of herbivores under ocean warming, causing energy depletion and mortality. Sci Total Environ. 2018;639: 360–366. doi:10.1016/j.scitotenv.2018.05.161

15.	Borell EM, Steinke M, Fine M. Direct and indirect effects of high pCO2 on algal grazing by coral reef herbivores from the Gulf of Aqaba (Red Sea). Coral Reefs. 2013;32: 937–947. doi:10.1007/s00338-013-1066-5

16.	Duarte C, López J, Benítez S, Manríquez PH, Navarro JM, Bonta CC, et al. Ocean acidification induces changes in algal palatability and herbivore feeding behavior and performance. Oecologia. 2016;180: 453–462. doi:10.1007/s00442-015-3459-3

17.	Gao G, Clare AS, Chatzidimitriou E, Rose C, Caldwell G. Effects of ocean warming and acidification, combined with nutrient enrichment, on chemical composition and functional properties of Ulva rigida. Food Chem. 2018;258: 71–78. doi:10.1016/j.foodchem.2018.03.040

18.	Gao G, Clare AS, Rose C, Caldwell GS. Ulva rigida in the future ocean: potential for carbon capture, bioremediation and biomethane production. GCB Bioenergy. 2018;10: 39–51. doi:10.1111/gcbb.12465

19.	Chen B, Lin L, Ma Z, Zhang T, Chen W, Zou D. Carbon and nitrogen accumulation and interspecific competition in two algae species, Pyropia haitanensis and Ulva lactuca, under ocean acidification conditions. Aquac Int. 2019; 721–733. doi:10.1007/s10499-019-00360-y

20.	Liu C, Zou D. Responses of elevated CO2 on photosynthesis and nitrogen metabolism in Ulva lactuca (Chlorophyta) at different temperature levels. Mar Biol Res. 2015;11: 1043–1052. doi:10.1080/17451000.2015.1062520

21.	Pavia H, Cervin G, Lindgren A, Åberg P. Effects of UV-B radiatio on phlorotannins in t Ascophyllum nodos. Mar Ecol Prog Ser. 1997;157: 139–146.

22.	Pavia H, Toth GB. Inducible Chemical Resistance to Herbivory in the Brown Seaweed Ascophyllum nodosum. Ecology. 2000;81: 3212–3225.

23.	Cruz-Rivera E, Hay ME. Can quantity replace quality? Food choice, compensatory feeding, and fitness of marine mesograzers. Ecology. 2000;81: 201–219. doi:10.1890/0012-9658(2000)081[0201:CQRQFC]2.0.CO;2

24.	Toth GB, Langhamer O, Pavia H. Inducible and constitutive defenses of valuable seaweed tissues: Consequences for herbivore fitness. Ecology. 2005;86: 612–618. doi:10.1890/04-0484

25.	Gutow L, Rahman MM, Bartl K, Saborowski R, Bartsch I, Wiencke C. Ocean acidification affects growth but not nutritional quality of the seaweed Fucus vesiculosus (Phaeophyceae, Fucales). J Exp Mar Bio Ecol. 2014. doi:10.1016/j.jembe.2014.01.005

26.	Poore AGB, Graba-Landry A, Favret M, Sheppard Brennand H, Byrne M, Dworjanyn SA. Direct and indirect effects of ocean acidification and warming on a marine plant-herbivore interaction. Oecologia. 2013;173: 1113–1124. doi:10.1007/s00442-013-2683-y

27.	Cruz-Rivera E, Hay ME. Macroalgal traits and the feeding and fitness of an herbivorous amphipod: The roles of selectivity, mixing, and compensation. Mar Ecol Prog Ser. 2001;218: 249–266. doi:10.3354/meps218249

28.	Bibby R, Cleall-Harding P, Rundle S, Widdicombe S, Spicer J. Ocean acidification disrupts induced defences in the intertidal gastropod Littorina littorea. Biol Lett. 2007;3: 699–701. doi:10.1098/rsbl.2007.0457

29.	Young CS, Lowell A, Peterson B, Gobler CJ. Ocean acidification and food limitation combine to suppress herbivory by the gastropod Lacuna vincta. Mar Ecol Prog Ser. 2019;627: 83–94. doi:10.3354/meps13087

30.	Schiel DR, Wood SA, Dunmore RA, Taylor DI. Sediment on rocky intertidal reefs: Effects on early post-settlement stages of habitat-forming seaweeds. J Exp Mar Bio Ecol. 2006;331: 158–172. doi:10.1016/j.jembe.2005.10.015

31.	Watt CA, Scrosati RA. Regional consistency of intertidal elevation as a mediator of seaweed canopy effects on benthic species richness, diversity, and composition. Mar Ecol Prog Ser. 2013;491: 91–99. doi:10.3354/meps10521

32.	Al-Janabi B, Kruse I, Graiff A, Winde V, Lenz M, Wahl M. Buffering and amplifying interactions among OAW (ocean acidification & warming) and nutrient enrichment on early life-stage Fucus vesiculosus L. (phaeophyceae) and their carry over effects to hypoxia impact. PLoS One. 2016;11: 1–18. doi:10.1371/journal.pone.0152948

33.	Surif MB, Raven JA. Exogenous inorganic carbon sources for photosynthesis in seawater by members of the Fucales and the Laminariales (Phaeophyta): ecological and taxonomic implications. Oecologia. 1989;78: 97–105. doi:10.1007/BF00377203

34.	Hepburn CD, Pritchard DW, Cornwall CE, Mcleod RJ, Beardall J, Raven JA, et al. Diversity of carbon use strategies in a kelp forest community: Implications for a high CO2 ocean. Glob Chang Biol. 2011;17: 2488–2497. doi:10.1111/j.1365-2486.2011.02411.x

35.	Werner FJ, Graiff A, Matthiessen B. Temperature effects on seaweed-sustaining top-down control vary with season. Oecologia. 2016;180: 889–901. doi:10.1007/s00442-015-3489-x

36.	Cardoso PG, Grilo TF, Dionísio G, Aurélio M, Lopes AR, Pereira R, et al. Short-term effects of increased temperature and lowered pH on a temperate grazer-seaweed interaction (Littorina obtusata/Ascophyllum nodosum). Estuar Coast Shelf Sci. 2017;197: 35–44. doi:10.1016/j.ecss.2017.08.007

37.	Hammill E, Johnson E, Atwood TB, Harianto J, Hinchliffe C, Calosi P, et al. Ocean acidification alters zooplankton communities and increases top-down pressure of a cubozoan predator. Glob Chang Biol. 2018;24: e128–e138. doi:10.1111/gcb.13849

38.	Robbins L., Hansen ME, Kleypas JA, Meylan SC. CO2calc- A user-friendly carbon calculator for Windows , Mac OS X , and iOS ( iPhone ) Florida Shelf Ecosystems Response to Climate Change Project CO2calc : A User-Friendly Seawater Carbon Calculator for Windows , Mac OS X , and iOS ( iPhone ). 2010.

39.	van Alstyne KL. Comparison of three methods for quantifying brown algal polyphenolic compounds. J Chem Ecol. 1995;21: 45–58. doi:10.1007/BF02033661

40.	Peterson CH, Renaud PE. Analysis of feeding preference experiments. Oecologia. 1989;80: 82–86. doi:10.1007/BF00789935

41.	Hickman RW, Illingworth J. Condition cycle of the green-lipped mussel Perna canaliculus in New Zealand. Mar Biol. 1980;60: 27–38. doi:10.1007/BF00395603

42.	Ober GT, Thornber CS. Divergent responses in growth and nutritional quality of coastal macroalgae to the combination of increased pCO2 and nutrients. Mar Environ Res. 2017;131: 69–79. doi:10.1016/j.marenvres.2017.09.003

43.	Takolander A, Cabeza M, Leskinen E. Seasonal interactive effects of pCO2 and irradiance on the ecophysiology of brown macroalga Fucus vesiculosus L. Eur J Phycol. 2019;54: 380–392. doi:10.1080/09670262.2019.1572226

44.	Graiff A, Bartsch I, Ruth W, Wahl M, Karsten U. Season exerts differential effects of ocean acidification and warming on growth and carbon metabolism of the seaweed fucus vesiculosus in the Western Baltic Sea. Front Mar Sci. 2015;2. doi:10.3389/fmars.2015.00112

45.	Kinnby A, Jonsson PR, Ortega-martinez O, Töpel M, Pavia H, Pereyra RT, et al. Combining an Ecological Experiment and a Genome Scan Show Idiosyncratic Responses to Salinity Stress in Local Populations of a Seaweed Projection of Future Salinity. 2020;7: 1–12. doi:10.3389/fmars.2020.00470

46.	Pretzsch H, Biber P, Schütze G, Uhl E, Rötzer T. Forest stand growth dynamics in Central Europe have accelerated since 1870. Nat Commun. 2014;5: 1–10. doi:10.1038/ncomms5967

47.	Pretzsch H, Biber P, Schütze G, Kemmerer J, Uhl E. Wood density reduced while wood volume growth accelerated in Central European forests since 1870. For Ecol Manage. 2018;429: 589–616. doi:10.1016/j.foreco.2018.07.045

48.	Bindoff, N.L., J. Willebrand, V. Artale, A, Cazenave, J. Gregory, S. Gulev, K. Hanawa, C. Le Quéré, S. Levitus, Y. Nojiri, C.K. Shum LDT and AU. Observations: Oceanic Climate Change and Sea Level. Changes.

49.	Hawkins SJ, Sugden HE, Mieszkowska N, Moore PJ, Poloczanska E, Leaper R, et al. Consequences of climate-driven biodiversity changes for ecosystem functioning of north European rocky shores. Mar Ecol Prog Ser. 2009;396: 245–259. doi:10.3354/meps08378

50.	Fernández PA, Roleda MY, Hurd CL. Effects of ocean acidification on the photosynthetic performance, carbonic anhydrase activity and growth of the giant kelp Macrocystis pyrifera. Photosynth Res. 2015;124: 293–304. doi:10.1007/s11120-015-0138-5

51.	Young CS, Gobler CJ. Ocean acidification accelerates the growth of two bloom-forming macroalgae. PLoS One. 2016;11: 1–21. doi:10.1371/journal.pone.0155152

52.	Kawamitsu Y, Boyer JS. Photosynthesis and carbon storage between tides in a brown alga, Fucus vesiculosus. Mar Biol. 1999;133: 361–369. doi:10.1007/s002270050475

53.	Robinson EA, Ryan GD, Newman JA. A meta-analytical review of the effects of elevated CO 2 on plant-arthropod interactions highlights the importance of interacting environmental and biological variables. New Phytol. 2012;194: 321–336. doi:10.1111/j.1469-8137.2012.04074.x

54.	Holopainen JK, Virjamo V, Ghimire RP, Blande JD, Julkunen-Tiitto R, Kivimäenpää M. Climate Change Effects on Secondary Compounds of Forest Trees in the Northern Hemisphere. Front Plant Sci. 2018;9: 1–10. doi:10.3389/fpls.2018.01445

55.	Arnold T, Mealey C, Leahey H, Miller AW, Hall-Spencer JM, Milazzo M, et al. Ocean acidification and the loss of phenolic substances in marine plants. PLoS One. 2012;7: 1–10. doi:10.1371/journal.pone.0035107
