# Supplementary material for: Ocean acidification decreases grazing pressure but alters morphological structure in a dominant coastal seaweed
Source: PLoS One. 2021 Jan 28;16(1):e0245017. doi: 10.1371/journal.pone.0245017 (PMC7842949; doi:10.1371/journal.pone.0245017)
Supplement: Attachment — Submitted filename: Response to Reviewers.docx [file pone.0245017.s004.docx]

**Response to reviewers:**

Reviewer #1: Kinnby et al. have added more details to the method section and cleared up other uncertainties as requested. I just have some minor suggestions for further improvement of the article. I think with these edits the manuscript will be ready for publication.

14: insert “projected to be 0.4 units lower..”

Response: The word lower has been inserted.

46: recommend changing “some” to “many” or even most

Response: Some has been changed to most.

66: insert pCO2 at the end of the sentence to give reference to the concentrations you are talking about

Response: pCO_2_ has been added to the end of the sentence.

72: here and a couple other places, OA is spelled out

Response: Thank you for pointing this out, this has been changed here and throughout the manuscript.

87: remove “very”

Response: Very has been removed from the sentence.

88: since they may not be carbon limited

Response: “Since they may not be carbon limited” has been added to the end of the sentence.

117: you should report the light levels if measured and type of light? LED? Natural? This is important since you discuss photosynthetic implications

Response: The experiment was performed in a greenhouse with natural lighting (natural light cycle 18:6 h, L:D).

128: space between number and units

Response: A space has been added.

141: change sd to SD

Response: sd has been changed to SD

176: add parentheses after SD & change all sd to SD

Response: A parentheses has been added and sd has been changed to SD throughout the manuscript.

251-252: Combine these two sentences…”The stable carbon isotope content of F. vesiculosus was significantly reduced to -13% when exposed to elevated pCO2.

Response: These two sentences have been merged to one.

276-278: reword, “which” is used twice in this sentence

Response: This sentence has been modified.

276-281: These last sentences could use some work, it is difficult to read. Maybe switch the last one around, “While the condition index increased…there was no change in preference and consumption actually decreased”

Response: These last sentences have been reworked.

291: But since you used genetically identical samples, how does this new information add to our existing understanding?

Response: This sentence has been altered for clarity. We want to clarify to the reviewer that we used the same individual in the treatment and its control, but there were not genetically identical samples for the entire experiment.

295: See Guenther et al 2017 “Macroalgal spore dysfunction” I believe they show weakened attachment strength – which could add to your story

Response: Thank you for bringing this article to our attention, we have added it to the text.

325: CCM – write out in full first time

Response: “carbon dioxide-concentrating mechanism” has been added to the sentence

327: Species names should be written out in full at the beginning of a sentence

Response: The full species name has been written out.

371: reword – suggest “Reduced consumption for the herbivore…”

Response: This has been reworded.

373: Was it really unanticipated?

Response: Yes, we weren’t expecting to find that the seaweeds would break more easily after exposure to elevated levels of pCO2. To our knowledge the literature on fleshy algae is pointing towards a positive effect of OA on these species since it generally enhances growth. As far as we know this is the first study that suggests that despite OA having a positive effect on growth there is a synergistic effect resulting in the algae becoming more vulnerable. Based on the available literature we did not expect to find this effect, and so it was unexpected for us. We appreciate that the reviewer brought our attention to Guenther et al. (2017) who suggest a similar pattern for spore attachment in red seaweeds.

Reviewer #2: (No Response)
